# Association between left ventricular mechanics and diffuse myocardial fibrosis in patients with repaired Tetralogy of Fallot: a cross-sectional study

**DOI:** 10.1186/s12968-017-0410-2

**Published:** 2017-12-11

**Authors:** Christopher M. Haggerty, Jonathan D. Suever, Arichanah Pulenthiran, Abba Mejia-Spiegeler, Gregory J. Wehner, Linyuan Jing, Richard J. Charnigo, Brandon K. Fornwalt, Mark A. Fogel

**Affiliations:** 1 Department of Imaging Science and Innovation, Geisinger, 100 North Academy Avenue, Danville, PA 17822-4400 USA; 2 Biomedical and Translational Informatics Institute, Geisinger, Danville, PA USA; 3 Department of Biomedical Engineering, University of Kentucky, Lexington, KY USA; 4 Department of Biostatistics, University of Kentucky, Lexington, KY USA; 5 Department of Radiology, Geisinger, Danville, PA USA; 6 Division of Cardiology, Children’s Hospital of Philadelphia, Philadelphia, PA USA

**Keywords:** Tetralogy of Fallot, T1 mapping, Strain, DENSE, MOLLI

## Abstract

**Background:**

Patients with repaired tetralogy of Fallot (TOF) have progressive, adverse biventricular remodeling, leading to abnormal contractile mechanics. Defining the mechanisms underlying this dysfunction, such as diffuse myocardial fibrosis, may provide insights into poor long-term outcomes. We hypothesized that left ventricular (LV) diffuse fibrosis is related to impaired LV mechanics.

**Methods:**

Patients with TOF were evaluated with cardiac magnetic resonance in which modified Look-Locker (MOLLI) T1-mapping and spiral cine Displacement encoding (DENSE) sequences were acquired at three LV short-axis positions. Linear mixed modeling was used to define the association between regional LV mechanics from DENSE based on regional T1-derived diffuse fibrosis measures, such as extracellular volume fraction (ECV).

**Results:**

Forty patients (26 ± 11 years) were included. LV ECV was generally within normal range (0.24 ± 0.05). For LV mechanics, peak circumferential strains (−15 ± 3%) and dyssynchrony indices (16 ± 8 ms) were moderately impaired, while peak radial strains (29 ± 8%) were generally normal. After adjusting for patient age, sex, and regional LV differences, ECV was associated with log-adjusted LV dyssynchrony index (β = 0.67) and peak LV radial strain (β = −0.36), but not LV circumferential strain. Moreover, post-contrast T1 was associated with log-adjusted LV diastolic circumferential strain rate (β = 0.37).

**Conclusions:**

We observed several moderate associations between measures of fibrosis and impaired mechanics, particularly the LV dyssynchrony index and peak radial strain. Diffuse fibrosis may therefore be a causal factor in some ventricular dysfunction in TOF.

## Background

Early surgical repair of tetralogy of Fallot (TOF) results in 20-year survival rates over 90%, but mortality rates increase substantially 25 years after surgery, mostly from cardiac causes [1]. Most patients have right ventricular (RV) volume overload stemming from chronic pulmonary regurgitation, and adverse ventricular remodeling, such as RV dilation, is common. This remodeling may contribute to the development of systolic dysfunction of both the RV and left ventricle (LV), as quantified by ejection fraction (EF) [2, 3], or more complex descriptors of cardiac mechanics, such as strains and dyssynchrony [4–6]. Systolic dysfunction is associated with poor outcomes, such as sudden death, sustained ventricular tachycardia, or exercise intolerance [2, 3, 7]. Understanding of the mechanisms underlying the development of this dysfunction is incomplete.

Adverse ventricular remodeling may include diffuse myocardial fibrosis; for example, remodeling may cause a fibrotic expansion of the extracellular volume fraction (ECV) [8]. In fact, recent studies have identified increased myocardial ECV in patients with TOF using T1 mapping cardiovascular magnetic resonance imaging (CMR) [9, 10]. One of those studies additionally identified elevated LV ECV as a risk factor for ventricular arrhythmia [10].

The presence of both diffuse myocardial fibrosis and impaired ventricular mechanics in patients with repaired TOF may be associated with each other through physical interaction. That is, the stiffening effects of fibrosis on the myocardium may impair cardiac function [11], or the loss of myofibrils through processes leading to fibrosis may alter myocardial mechanics. In fact, recent studies have demonstrated weak-to-moderate associations between ECV and myocardial strains, strain rates, or dyssynchrony in hypertensive patients with hypertrophy, patients with heart failure, as well as subjects from the multi-ethnic study of atherosclerosis (MESA) cohort [12–14]. However, this association has not been studied in TOF, which represents a unique anatomic and physiologic setting. We hypothesized that LV diffuse fibrosis is associated with impaired LV mechanics in patients with repaired TOF.

## Methods

### Patient selection

The study protocol was approved by the institutional review board at The Children’s Hospital of Philadelphia, and informed consent for research was obtained from all patients. Between December 2012 and July 2015, a modified Look-Locker (MOLLI) T1 mapping sequence [15, 16] and spiral cine Displacement Encoding with Stimulated Echoes (DENSE) sequence [17–19] were both acquired in 43 patients with repaired TOF undergoing a clinical CMR evaluation.

### Image acquisition and analysis

All images were acquired using a 1.5-T Siemens Avanto (Siemens Healthineers, Erlangen, Germany) CMR system. For each subject, the imaging protocol included cine balanced steady-state free precession (bSSFP) images, 2D phase contrast velocity, phase-sensitive inversion recovery, spiral cine DENSE, and MOLLI acquired both before and 15–20 min after administration of gadolinium contrast (gadopentetate dimeglumine, 0.2 mmole/kg, Bayer Health Care, Whippany New Jersey, USA). The MOLLI and DENSE acquisitions were co-localized at 3 ventricular short-axis locations (basal, mid-ventricular, and apical), such that cardiac mechanics and diffuse myocardial fibrosis could be interrogated over the same regions and directly compared. Details for the acquisition and post-processing for each specific sequence are as follows.

#### MOLLI

For MOLLI, the pre-contrast acquisition scheme was 5(3)2 or 5(3)3, while the post-contrast scheme was 4(1)3(1)2 or 4(1)2(1)2. The pulse sequence parameters included: Initial TI 90–120 ms, TE 1.0–1.3 ms, TR 3.6–12.9 ms, flip angle 35°, resolution 1.0–1.9 × 1.0–1.9 mm, and slice thickness 6–8 mm. Motion correction and curve fitting for T1 computation were performed online automatically during image reconstruction.

LV borders were manually contoured on MOLLI T1 images for fibrosis analysis, using custom MATLAB software (The Mathworks, Natick, Massachusetts, USA). Care was taken to restrict the region of interest to the mid-myocardial layer to minimize contamination of T1 signal at blood or air interfaces. The anterior RV insertion point was manually marked to annotate LV segments based on the standard 16-segment model. Native and post-contrast T1 values were quantified for the myocardium and blood pool for each region (slice) and each segment. From these measures, gadolinium partition coefficient (λ_Gd_) was computed:

$$ {\lambda}_{Gd}=\frac{\frac{1}{T_{1\  myo\  with\  Gd}}-\frac{1}{T_{1\  myo\  with out\  Gd}}}{\frac{1}{T_{1\  blood with\  Gd}}-\frac{1}{T_{1\  blood with out\  Gd}}}. $$

Hematocrit measurements were not available; however, hematocrit was estimated based on a validated relationship with the native T1 of blood [20]. Hence, the ‘synthetic’ ECV was computed as:

$$ ECV={\uplambda}_{Gd}\left(1- Hematocrit\right). $$

#### DENSE

DENSE acquisition parameters included: 6 spiral interleaves (2 interleaves acquired per temporal frame); 0.10 cycles/mm in-plane encoding frequency, with simple encoding [18]; 0.08 cycles/mm through-plane de-phasing frequency [21]; Complementary spatial modulation of magnetization (CSPAMM) echo suppression [19]; TR 15 ms (30 ms temporal resolution without view sharing); TE 1.08 ms; flip angle 20°; resolution 1.8–2.8 × 1.8–2.8 mm; slice thickness 8 mm.

DENSE images were analyzed in *DENSEanalysis* [22], an open-source MATLAB program. Endocardial and epicardial boundaries were manually delineated on the DENSE magnitude images, followed by automatic phase unwrapping, spatial smoothing, and temporal fitting of phase displacement data [23], and calculation of LV mechanics. These measures included peak myocardial strains (radial and circumferential), peak circumferential strain rates (systolic and diastolic), and a dyssynchrony index (DI), each of which were quantified for each region (base, mid-ventricle, apex), in addition to a global mean value. To quantify dyssynchrony, the mean contraction delay was computed for each segment by comparing the circumferential strain curves for each element to a patient-specific reference curve with a cross-correlation analysis [24]. The segmental delays were circumferentially smoothed with a cubic spline interpolant, and DI for each region was computed as the standard deviation of the segmental delays [4]. Data were partitioned to the 16-segment model, based on the manual definition of the anterior RV insertion point, so peak strain results were also defined at the segmental level, matching the T1 data. The reproducibility of segmental strain rates has not been demonstrated, so those measures were only used to derive regional and global strain rates, and segmental strain rates were not included.

#### bSSFP

A contiguous short-axis stack of bSSFP images spanning the ventricles was also acquired. The pulse sequence parameters included: TR 2.7–9.0 ms, TE 1.2–4.5 ms, flip angle 15–90°, resolution 1.0–1.9 × 1.0–1.9 mm, slice thickness 6.5–10 mm. These images were manually segmented to quantify LV and RV volumes and ejection fraction and LV mass based on a sum of slices technique. Volumes and mass were indexed to body surface area [25].

#### 2D phase contrast (velocity)

Pulmonary regurgitation was quantified using a single through-plane encoded phase contrast velocity encoded image positioned in the main pulmonary artery. The velocity encoding for these acquisitions ranged from 150 to 350 cm/s (adjusted to avoid phase aliasing). Other parameters included TR 11.4–13.3 ms, TE 2.5–3.0 ms, flip angle 25°, resolution 1.0–1.8 × 1.0–1.8 mm, slice thickness 4–5 mm. These images were manually segmented using custom MATLAB software (The Mathworks).

#### Phase-sensitive inversion recovery

Late gadolinium enhancement (LGE) was clinically evaluated using phase-sensitive inversion recovery images. For each subject, LGE was qualified as “present” or “absent” based on the clinical interpretation.

### Intra- and inter-observer reproducibility

Segmentation of the MOLLI T1 images was repeated 2 months following the initial analysis by the primary analyst, and independently by a second analyst to quantify intra- and inter-observer reproducibility, respectively. The mean coefficient of variation (CoV) and the 95% limits of agreement [26] were quantified. The mean CoV was computed as:

$$ \frac{\sum_{i=1}^N\left[ St. Dev.{\left({X}_{Obs.1}\ {X}_{Obs.2}\right)}_i\right]}{\left|{\sum}_{i=1}^N\left[{\left(\frac{X_{Obs.1}+{X}_{Obs.2}}{2}\right)}_i\right]\right|} $$

### Statistical analysis

Analysis was performed in R (version 3.4.0; R Foundation for Statistical Computing, Vienna, Austria) [27]. Data are presented as mean ± standard deviation. Z-scores for ventricular volumes, mass, and EF were computed based on population data assumed from Alfakih et al. [28] Pearson correlation was used to detect simple associations between strain/T1 derived measures and ventricular volumes, LV mass, and LV/RV EF. Linear mixed models [29] were fit to capture the mean association of regional (base, mid-ventricle, or apex) or segmental LV mechanics with T1-derived measures, while accounting for effects of sex, age, measurement region or segment, and within-subjects repeated measures (e.g., multiple regions or segments per patient). Repeated measures for the primary regional analysis were modeled using an unstructured correlation matrix. For the segmental analysis, a compound symmetric correlation matrix was assumed to ensure model convergence. Continuous outcome and independent variables were standardized for model fitting to report standardized β coefficients. Outcome variables were log transformed, as appropriate, to adjust for heteroscedasticity or non-normality. The statistical significance level was set at 0.05, with a Benjamini-Hochberg adjustment for multiple comparisons of each outcome variable [30]. Preliminary power analysis [31], assuming a linear correlation coefficient of 0.30, demonstrated that 84 samples were required to achieve 80% power.

## Results

### Study group

From the original 43 patients, two were excluded for either poor DENSE image quality (*n* = 1) or errors with the reconstruction of T1 images (*n* = 1). An additional patient was excluded because of DENSE and MOLLI image slice mis-registration, leaving a total of 40 subjects in the final study group. An additional eight DENSE slices (4 base, 4 apex) from seven different subjects were also excluded for poor quality. Because of the need to compute a ventricular reference curve for the dyssynchrony analysis, the dyssynchrony index was not computed for these seven subjects. However, the remaining data for these subjects were included. Demographic details as well as ventricular volume indices, EF, and LV mass indices for these subjects are provided in Table 1. The RVs on average were severely dilated (Z = 4.6) with reduced EF (Z = −3.1), while the LVs had mostly normal chamber volume (Z = −0.6) and myocardial mass (Z = −0.7), but impaired EF (Z = −2.0). These structural and global functional characteristics are generally consistent with contemporary findings from the INDICATOR study [3].

**Table 1:** Demographic and ventricular volume details for 40 patients with repaired TOF

	Result	Z-score
Age [years]	25.5 ± 10.5	
Sex	Male: 23; Female: 17	
BSA [m^2^]	1.7 ± 0.3	
LV EDV_i_ [mL/ m^2^]	72.8 ± 13.3	−0.6 ± 1.1
LV ESV_i_ [mL/ m^2^]	32.8 ± 7.3	
LV Mass_i_ [g/ m^2^]	53.1 ± 8.1	−0.7 ± 0.9
LV EF [%]	54.9 ± 7.3	−2.0 ± 1.6
LV LGE (% of pts)	37.5	
RV EDV_i_ [mL/ m^2^]	145.1 ± 44.9	4.6 ± 3.1
RV ESV_i_ [mL/ m^2^]	82.0 ± 36.7	
RV EF [%]	44.1 ± 10.2	−3.1 ± 2.5
Pulmonary Regurgitation [%]	31.9 ± 19.2	

*BSA* body surface area, *EDV*_*i*_ indexed end-diastolic volume, *EF* ejection fraction, *ESV*_*i*_ indexed end-systolic volume, *LV* left ventricular, *Mass*_*i*_ indexed myocardial mass, *RV* right ventricular

Most patients in this study (62.5%) were negative for LGE (Table 1). Of those with LGE reported, the predominant finding (in 73%) was a small region of enhancement at the inferior RV hinge point.

### Diffuse fibrosis and strain findings

**Fig. 1:**
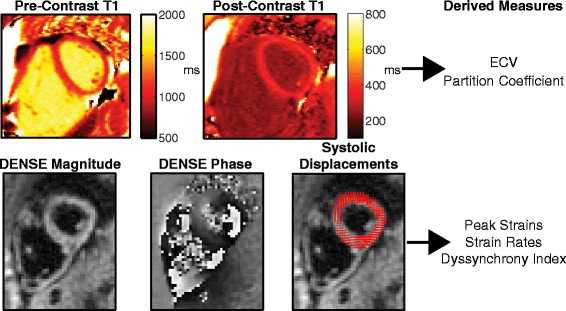
Example MOLLI T1 and DENSE images. Top row shows Pre- and Post-Contrast MOLLI T1 images for a representative patient. Bottom row shows DENSE magnitude and phase images for the same patient and same slice near end-systole, as well as the resulting displacement vectors at the same instant. The derived measures from each imaging sequence are listed for clarity

**Fig. 2:**
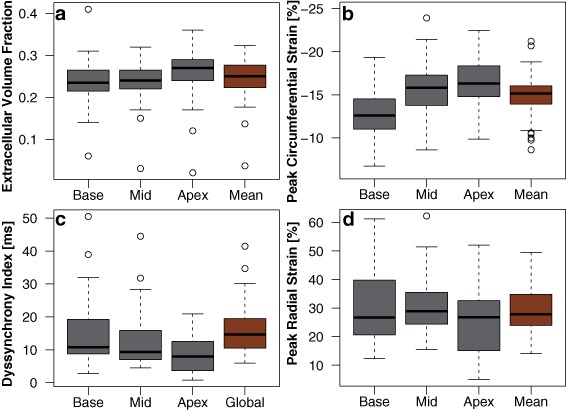
Box Plots of Circumferential and Radial Strains and ECV Results. Measures of **a**) extracellular volume fraction (ECV), **b**) peak circumferential strain, **c**) dyssynchrony index (DI), and **d**) peak radial strain represented as boxplots. Data are shown for each region (Base, Mid-ventricle, and Apex) as well as the global average (“Mean”) across slices

Representative T1 and DENSE images are shown in Fig. 1, and summary measures are presented in Table 2. Figure 2 represents the measured regional values of ECV, DI, and circumferential and radial strains, as well as global (“mean”) values. LV ECV (Fig. 2a) was generally within normal ranges, although mean values for seven patients (18%) were greater than 0.28, which has previously been reported as the upper bound of the normal range [10]. LV ECV was moderately associated with age (*r* = 0.35, *p* = 0.03), but not with LV or RV EF, LV mass index, LV or RV end-diastolic volume indices, or pulmonary regurgitant fraction (data not shown). Additionally, mean ECV was not different between patients based on the presence or absence of LGE (data not shown).

**Table 2:** Primary imaging findings

**T1**	
Native T1 [ms]	952 ± 73
Post-Contrast T1 [ms]	414 ± 54
λ_Gd_	0.458 ± 0.051
ECV	0.245 ± 0.051
**Mechanics**	
Peak Circumferential Strain [%]	−15.0 ± 2.8
Peak Radial Strain [%]	29.3 ± 8.3
DI [ms]	16.1 ± 8.4
Systolic Circumferential SR [%/s]	−93 ± 17
Diastolic Circumferential SR [%/s]	127 ± 35

*λ*_*Gd*_ gadolinium partition coefficient; *ECV* extracellular volume fraction, *SR* strain rate, *DI* dyssynchrony index

Peak LV circumferential strain (Fig. 2b) demonstrated a gradient of increasing magnitude from base to apex, which is consistent with normal LV function [32]. However, mean values were at the low (magnitude) end of the normal range, with 12 patients (31%) having strain magnitudes below the normal range (i.e., > −14.4%). Only two of these 12 patients also had an elevated mean LV ECV. This moderate impairment in LV circumferential strain mirrored the impairment in LV EF, as the two measures were significantly correlated (*r* = −0.54, *p* < 0.001), but mean LV circumferential strain was not associated with LV mass, LV/RV volumes, RV EF, or pulmonary regurgitation, and did not differ between patients with or without LGE. LV dyssynchrony was moderately elevated from the normal mean (DI = 16.1 ± 8 ms; Fig. 2c), and seven patients (21% with measured DI) had a value above the normal range (>21.1 ms) [4]. Only three of these seven patients also had reduced mean LV circumferential strain. Finally, mean LV radial strains were generally within the normal range (Fig. 2d), and did not differ between patients with or without LGE (data not shown).

**Fig. 3:**
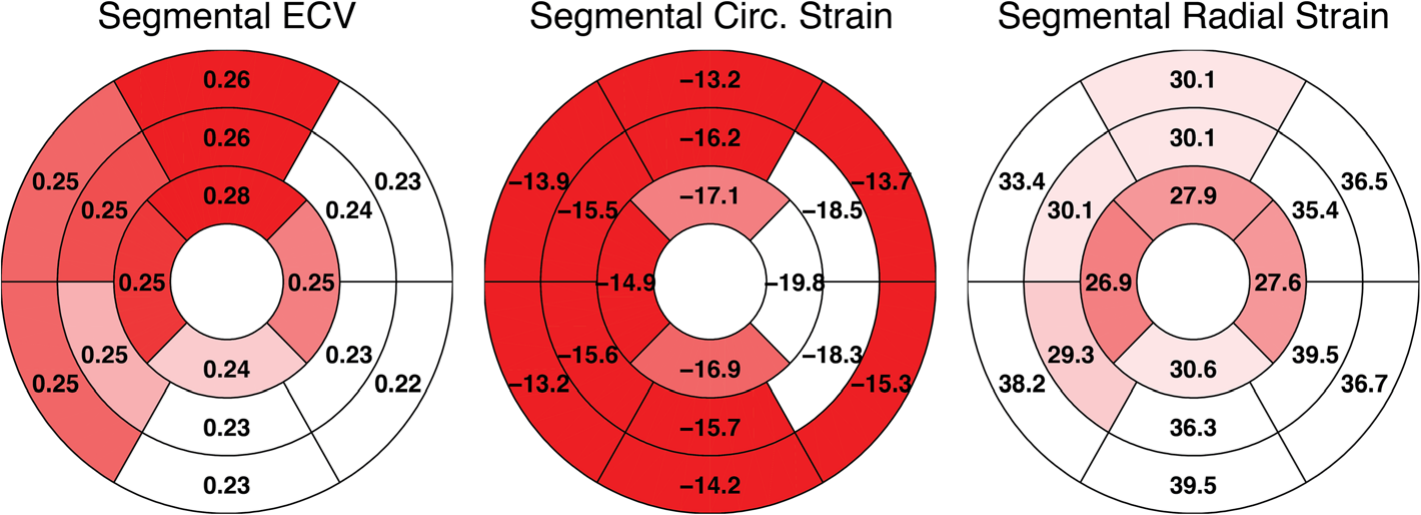
Bullseye Plots for Segmental Means. ECV (left), circumferential (middle) and radial strain (right) data presented with respect to the standard 16-segment left ventricular (LV) model. The mean of each segment is overlaid on the segment and graphically represented by the color relative to the other segments

At the segmental level, Fig. 3 presents the values for LV ECV, LV circumferential strain, and LV radial strain for the standard 16-segment LV model. LV ECV values tended to be higher in anterior and septal segments than the posterior and free wall, although the overall spatial variation was low (standard deviation of the segment means, *SD* = 0.02). LV circumferential strains in the mid-ventricular and apical free wall segments were preserved; however, strain in other segments were considerably lower (*SD* = 2.0%). LV radial strain also demonstrated segmental heterogeneity (*SD* = 4.4%), with higher strains in the basal and mid-ventricular free wall compared with the apex and mid-ventricular septum.

### Association between fibrosis and mechanics

**Fig. 4:**
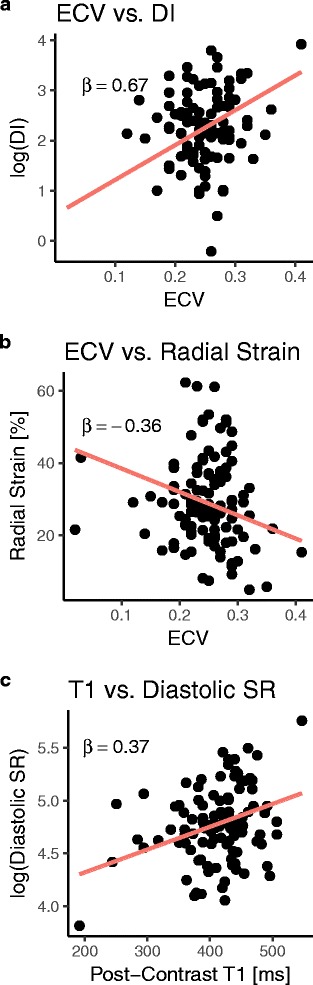
T1-Based Measures are Associated with LV Mechanics. Scatter plots demonstrating the regional multivariate models in which ECV is associated with **a**) the log-adjusted LV dyssynchrony index (DI) and **b**) peak LV radial strain, and **c**) post-contrast T1 predicts the log-adjusted LV diastolic circumferential strain rate. The standardized β coefficient of the primary independent variable is reported in each case

From mixed model analyses, T1-derived measures had the strongest association with regional LV DI (Table 3), as λ_Gd_, ECV, and post-contrast T1 time were all significantly associated with increased regional LV DI after adjusting for age, sex, and regional differences. In each case, the β coefficients of these associations ranged between 0.33 (absolute) and 0.67, with ECV having the largest individual coefficient (Fig. 4a). Moreover, four of the seven patients with elevated mean ECV also had an abnormal mean LV DI. All T1-derived measures were also significantly associated with reduced regional LV radial strain, with ECV again having the largest magnitude coefficient (β = −0.36; Fig. 4b). The other statistically significant models were post-contrast T1 being related with regional LV systolic (β = −0.30) and (log-transformed) diastolic circumferential strain rates (β = 0.37; Fig. 4c). There was also a trend with post-contrast T1 and regional LV circumferential strain (*p* < 0.05; Table 3), but this did not meet statistical significance thresholds after adjustment for multiple hypothesis testing. No associations were observed at the segmental level (Table 4).

**Table 3:** Results of mixed model analyses for LV regional measures

Outcome	T1-based predictor	Standardized β	Predictor *p*-value
Radial Strain	λ_Gd_	−0.33	<0.001 ^a^
Radial Strain	ECV	−0.36	0.002 ^a^
Radial Strain	Post-contrast T1	0.27	0.01 ^a^
Circumferential Strain	λ_Gd_	0.09	0.27
Circumferential Strain	ECV	0.11	0.31
Circumferential Strain	Post-contrast T1	−0.22	0.05
log(DI)	λ_Gd_	0.47	<0.001 ^a^
log(DI)	ECV	0.67	<0.001 ^a^
log(DI)	Post-contrast T1	−0.33	0.01 ^a^
Systolic Circumferential SR	λ_Gd_	0.15	0.09
Systolic Circumferential SR	ECV	0.13	0.26
Systolic Circumferential SR	Post-contrast T1	−0.30	0.014 ^a^
Diastolic Circumferential SR	λ_Gd_	−0.15	0.10
Diastolic Circumferential SR	ECV	−0.22	0.05
log(Diastolic Circumferential SR)	Post-contrast T1	0.37	<0.001 ^a^

*λ*_*Gd*_ gadolinium partition coefficient, *ECV* extracellular volume fraction, *SR* strain rate, *DI* dyssynchrony index

^a^ statistical significance retained after Benjamini-Hochberg adjustment

**Table 4:** Results of mixed model analyses for LV segmental measures

Outcome	T1-based predictor	Standardized β	Predictor *p*-value
Radial Strain	λ_Gd_	0.002	0.97
Radial Strain	ECV	0.007	0.90
Radial Strain	Post-contrast T1	0.04	0.48
Circumferential Strain	λ_Gd_	−0.04	0.33
Circumferential Strain	ECV	−0.03	0.55
Circumferential Strain	Post-contrast T1	0.05	0.44

*λ*_*Gd*_ gadolinium partition coefficient, *ECV* extracellular volume fraction

### Reproducibility

**Fig. 5:**
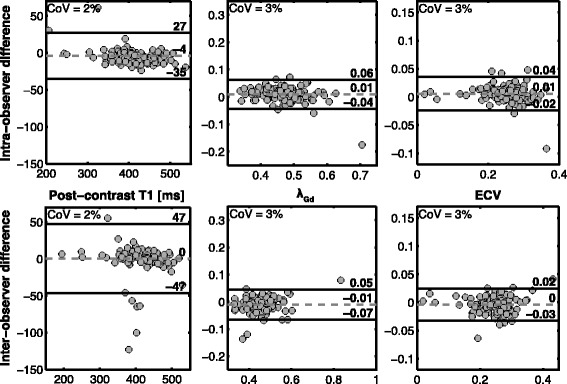
Reproducibility of T1 mapping. Bland-Altman comparisons of intra- (top) and inter-observer (bottom) analyses of T1 mapping data with respect to the quantification of post-contrast T1 (left), λ_Gd_ (middle), and ECV (right). In addition to the bias and limits of agreement, the mean coefficient of variation (COV) is reported for each plot

Consistent with previous studies [10], T1-derived measures were highly reproducible at a regional level on both intra- and inter-observer bases (Fig. 5). In all cases, the mean CoV was less than or equal to 3%, denoting tight limits of agreement relative to measurement means. The reproducibility of segmental-level values was similarly strong, with mean CoVs ≤5% (not shown).

## Discussion

Diffuse myocardial fibrosis and cardiac dysfunction of the LV have both been implicated as risk factors for adverse cardiac events, such as arrhythmia, in patients with repaired TOF [3, 10]. However, the extent to which these factors are linked—as has been shown in acquired heart disease [12–14, 33]—is unclear. Therefore, using MOLLI T1 mapping and DENSE CMR, we quantified markers of diffuse fibrosis and cardiac mechanics throughout the LV to elucidate the potential interplay between these characteristics for the first time in patients with repaired TOF. As hypothesized, we found associations at a regional level, indicating that T1-derived markers of fibrosis were related with LV dyssynchrony, peak LV radial strains, and LV circumferential strain rates. However, there was also evidence that components of LV function/dysfunction were independent of diffuse fibrosis, such as peak circumferential strains and ejection fraction. Collectively, these findings suggest that diffuse fibrosis is one of multiple drivers of cardiac dysfunction in TOF. The prognostic significance of these different factors will require further longitudinal study.

### LV fibrosis

From our findings, the overall LV myocardial burden of diffuse fibrosis in patients with repaired TOF was generally low, based on data from healthy controls in other studies [34, 35]. This finding is consistent with the results of Chen et al., who similarly reported a mean LV ECV of 0.24 ± 0.03 in a cohort of 84 TOF patients of similar mean age [10]. However, seven patients (18%) had an elevated ECV (>0.28), which was previously associated with increased arrhythmic risk [10]. Continued follow-up of our patients is needed to determine if a similar risk association is observed. Our findings do conflict with those of Broberg et al., who reported a slightly higher LV ECV (0.29 ± 0.03) in a smaller (*n* = 17) patient group [9]. The patients in that study were older (34 ± 12 years), which may explain this difference given the positive association we observed between ECV and age.

### LV function and fibrosis

Findings of reduced systolic LV function, including impaired myocardial strain, are common in patients with repaired TOF [4, 5, 36–39]; the fact that mean LV circumferential strain was moderately impaired in this study was not surprising. However, none of the T1-derived measures were associated with circumferential strain at either the regional or segmental levels, which was unexpected given that such associations were reported by Kuruvilla et al. [12] and Donekal et al. [14] in acquired heart diseases. This difference suggests a distinct etiology of impaired circumferential strain in the setting of repaired TOF, which is not fibrosis-related. Instead, these changes likely result from other mediating factors, such as surgical techniques [2] or septal shifting secondary to abnormal ventricular-ventricular interactions [36, 38]. Alternatively, the pattern of fibrosis may be such that myofibril contraction is affected to a much greater extent in the radial direction than in the circumferential direction.

The functional factor that demonstrated the strongest relationship with T1-derived measures of fibrosis was the LV dyssynchrony index. Findings of dyssynchrony in TOF are also common [4, 36, 40], and have been thought to be evidence of inter-ventricular interaction [4]. Our results suggest that diffuse fibrosis may be a primary mediator of the development of LV dyssynchrony in these patients. This linkage may help to explain the association between elevated LV ECV and increased risk of arrhythmia [10] through mechanisms such as electromechanical coupling [36, 41], but further study is warranted to definitively establish such connections. More generally, the prognostic significance of ventricular dyssynchrony in TOF has not been fully evaluated. A recent study found that dyssynchrony derived from CMR feature tracking did not predict longitudinal changes in LV or RV EF [5], although endpoints such as arrhythmias and mortality were not considered.

The remaining regional mechanical factors found to depend on T1-derived measures of fibrosis were LV radial strains and LV circumferential strain rates. Interestingly, the mean values for these measures were comparable to data for healthy controls from other studies [42–44], suggesting that they may serve as a compensatory mechanism to prevent more severe reductions in EF. The dependence on ECV, particularly for radial strain, is thus a potential cause for concern if the fibrotic expansion or cell atrophy [10] weakens that compensatory ability. Longitudinal follow-up, particularly with continued increase in ECV expansion, is needed to further evaluate these relationships.

### Limitations

This was a small cross-sectional study, so the ability to firmly establish causal relationships or long-term significance was limited. However, the use of CMR to measure all endpoints did ensure excellent reproducibility and reduced variance to enhance statistical power [45].

We did not quantify fibrosis or cardiac mechanics for the RV, which would be of obvious interest in repaired TOF, because of the generally insufficient resolution of the images for the thin wall of the RV. Future studies will leverage newer, higher resolution T1 mapping techniques [46] and higher resolution DENSE to better resolve the RV [44]. However, given the fact that LV diffuse fibrosis [10] and LV systolic dysfunction [3] have been implicated as predictors of poor outcomes in TOF, our focus on the LV is highly relevant.

Hematocrit data were not systematically available for all patients, necessitating the use of a “synthetic” hematocrit estimation to define ECV. This model may have produced inaccurate estimates for some patients, particularly younger individuals. While not the current gold standard for ECV estimation, it is important to note that the synthetic hematocrit model was derived and validated using a MOLLI sequence on 1.5 T Siemens scanners (inclusive of the Avanto model used for the present study), and demonstrated excellent agreement with the conventional measurement (95% limits of agreement of approximately ±4%) [20]. Given these findings, we feel its use, in the absence of hematocrit data, is justified. Moreover, associations of ECV and mechanics were generally similar to those of other T1-based endpoints, which did not require the synthetic hematocrit assumption, demonstrating the appropriateness of the ECV estimations.

No normal reference range for ECV was available from our laboratory, as is recommended for clinical reporting [47]. Differences in acquisition and analysis techniques may thus confound comparisons of absolute values to published reference ranges. However, as the primary objective of the study was to quantify associations with cardiac mechanics, this limitation had minimal effect on our primary findings.

Finally, no long-axis DENSE or T1 data were acquired to allow for the quantification of LV longitudinal strain. Previous studies have shown impairments in longitudinal strain in patients with TOF [36], so future studies should additionally explore the relationship of such impairments with fibrosis.

## Conclusion

The factors governing LV mechanics in patients with repaired TOF are complex. We found that elevated diffuse LV myocardial fibrosis was associated with regional LV dyssynchrony and peak radial strain, while decreasing post-contrast T1 was associated with abnormal circumferential strain rates. Extracellular matrix expansion and fibrotic remodeling thus potentially represent one of the mediating factors of LV function. However, other important components of LV function, such as regional circumferential strain, appeared to be impaired independent of fibrosis. Hence, while it is tempting to speculate that diffuse fibrosis may precede frank systolic dysfunction and provide early prognostic insight, it appears more likely that detailed characterization of both fibrosis and cardiac mechanics as part of routine surveillance of these patients is needed to fully capture their independent contributions.

## Abbreviations

bSSFP: Balanced steady state free precession
CMR: Cardiovascular magnetic resonance
CoV: Coefficient of Variation
DENSE: Displacement encoding with stimulated echoes
DI: Dyssynchrony index
ECV: Extracellular volume fraction
EF: Ejection fraction
LGE: Late gadolinium enhancement
LV: Left ventricle/left ventricular
MOLLI: Modified Look-Locker
RV: Right ventricle/right ventricular
TE: Echo time
TOF: Tetralogy of Fallot
TR: Repetition time
λ_Gd_: Gadolinium partition coefficient
